# caRBP-Pred: Leveraging Protein Language Models for the Prediction of Chromatin-Associated RNA-Binding Proteins

**DOI:** 10.34133/csbj.0060

**Published:** 2026-06-05

**Authors:** Qiang Sun, Feng Yang, Hao Sun, Huating Wang, Xiaona Chen

**Affiliations:** ^1^ Department of Orthopaedics and Traumatology, Li Ka Shing Institute of Health Sciences, The Chinese University of Hong Kong, Hong Kong, China.; ^2^ Center for Neuromusculoskeletal Restorative Medicine, Hong Kong SAR, China.; ^3^ Hangzhou Institute of Medicine, Chinese Academy of Sciences, Hangzhou, Zhejiang, China.; ^4^ Department of Bioinformatics, Warshel Institute for Computational Biology, School of Medicine, The Chinese University of Hong Kong, Shenzhen, Guangdong, China.

## Abstract

RNA-binding proteins (RBPs) play pivotal roles in cellular processes ranging from RNA metabolism to 3-dimensional genome organization. A distinct subset, chromatin-associated RBPs (caRBPs), binds directly to chromatin to function as transcriptional regulators. However, experimental identification of caRBPs using techniques such as chromatin immunoprecipitation sequencing and mass spectrometry is labor-intensive and costly. Existing computational tools for DNA-binding protein and RBP prediction often rely on outdated Gene Ontology annotations and fail to capture the unique characteristics of chromatin association. Here, we introduce caRBP-Pred, a deep learning framework that integrates a pre-trained protein language model with convolutional neural networks and bidirectional long short-term memory networks. By leveraging full-length protein sequences and evolutionary embeddings from ProtT5-XL, caRBP-Pred significantly outperforms existing DNA- and RNA-binding protein predictors. Application of our model to the mouse proteome identified 41 high-confidence caRBP candidates. Multidimensional validation using the COMPARTMENTS database and InterProScan confirmed that a proportion of these candidates possess experimentally verified chromatin-binding domains or high-confidence nuclear localization. Collectively, caRBP-Pred is the first computational tool specifically designed for caRBP prediction, offering a valuable resource for investigating the regulatory roles of caRBPs in chromatin-related function.

## Highlights

• caRBP-Pred is a novel deep learning framework that accurately predicts chromatin-associated RNA-binding proteins (caRBPs).
• The protein language model–convolutional neural network–bidirectional long short-term memory framework achieves competitive predictive performance in identifying caRBPs.
• caRBP-Pred identifies high-confidence mouse caRBP candidates, with a substantial portion validated by established chromatin localization data.
• Enriched motifs in chromatin-contact peptides reveal electrostatic and phase-separation mechanisms for chromatin recruitment.

## Introduction

RNA-binding proteins (RBPs) are well established as key regulators of gene expression, operating through diverse mechanisms. Their classical role in post-transcriptional regulation encompasses RNA alternative splicing, localization, translation, and stabilization [1–5]. More recently, RBPs have also been implicated in transcriptional regulation and 3-dimensional genome organization [6–11]. Thus, RBPs can profoundly influence a wide range of biological functions through multiple pathways. Consequently, a comprehensive analysis of RNA/DNA–RBP complexes and their molecular functions is essential for a deep understanding of these interactions. Recent studies have revealed that a wide variety of RBPs can bind to open chromatin and active chromatin regions including promoters and enhancers. These interactions influence transcription and/or mediate co-transcriptional processing, providing novel insights into the field of gene expression [6,7,12,13]. For example, QKI5, which is a chromatin-associated RBP (caRBP) identified in K562, 293T, and THP1 cells, can bind to chromatin independent of RNA or transcription factors and can function as a transcriptional activator to activate monocytic differentiation-associated genes [12]. Another caRBP, Dazl, colocalizes with PRC2 at the promoters of genes in mouse embryonic stem cells (mESCs) and is involved in the formation of phase-separated polycomb repressive complex condensates [14]. Currently, numerous experimental methods have been used to identify the DNA-binding sites of RBPs, such as chromatin immunoprecipitation sequencing [7], CUT&RUN, CUT&Tag, and mass spectrometry [2,7,11,12,15]. However, the identification of caRBPs using these methods is labor-intensive and costly, due to the complex experimental procedures involved. Moreover, they are limited in their capacity to identify multiple RBPs simultaneously.

Over the past decades, a suite of computational methods leveraging deep learning and machine learning has emerged to predict RBPs. These models are typically trained on protein or RNA primary sequences, often incorporating derived features such as physicochemical properties (e.g., hydrophobicity, polarity, and side-chain charge) and secondary structures (SSs). For example, tools like RBPPred and NAbind utilize physicochemical properties or electrostatic information with support vector machines (SVMs) to predict RBPs [16,17]. Other models, such as Deep-RBPPred, harness convolutional neural networks (CNNs) to enhance prediction accuracy [18]. Additionally, models like rBPDL integrate CNN–long short-term memory architectures with full-length protein inputs to further improve performance [19]. While protein language models (pLMs) have introduced rich evolutionary context and enable the effective prediction or classification of RBPs, a critical gap remains in the specialized prediction of caRBPs [20–23].

However, a critical distinction exists between generic DNA- and RNA-binding proteins (DRBPs) and caRBPs. While DRBPs are typically defined by their capacity to bind directly to specific DNA or RNA sequences, caRBPs represent a distinct functional subset. caRBPs associate with chromatin not only through direct nucleic acid binding but also potentially via interactions with histone modifications and chromatin-associated proteins or through phase-separation mechanisms [14]. Unlike classical DRBPs, caRBPs are characterized by their ability to bind chromatin in an RNA-independent manner to regulate transcription and 3-dimensional genome organization. Consequently, existing DRBP predictors (e.g., iDRBP_MMC, DeepDRBP-2L, and DeepMC-iNABP) face limitations: they rely on Gene Ontology annotations that may lag behind current knowledge, and they are not designed to capture the unique features of chromatin–caRBP interactions [24–26].

In this study, we developed caRBP-Pred, a pLM–CNN–bidirectional long short-term memory (BiLSTM) framework specifically designed to predict caRBPs in mice. By integrating high-dimensional embeddings from ProtT5-XL with CNN–BiLSTM architecture, our model effectively extracts complex functional features and significantly outperforms the performance of traditional sequence-based models. Leveraging a curated dataset of RNA-independent caRBPs from mESCs, we demonstrate that caRBP-Pred significantly improves the identification of these specialized regulators. To our knowledge, this is the first computational tool specifically designed for protein-level caRBP prediction, providing a reliable resource for future investigation of the regulatory roles of RBPs in chromatin-related function.

## Material and Methods

### Dataset construction and preprocessing

A comprehensive dataset was assembled comprising 403 caRBPs vs. 1,694 non-caRBPs, along with 1,532 chromatin-contact peptides vs. 14,937 negative peptides derived from mESCs, as previously reported [14]. To eliminate sequence redundancy, we utilized CD-HIT with similarity thresholds of 40% for proteins and 80% for peptides [27]. After filtering, the final dataset consisted of 349 caRBPs vs. 1,445 non-caRBPs and 822 chromatin-contact peptides vs. 10,384 negative peptides.

To handle variable lengths, protein and peptide sequences were truncated or zero-padded to fixed lengths of 2,000 and 50 residues, respectively. The total dataset was partitioned into an 80% training/validation set and a 20% independent held-out testing set using a random state of 42 for reproducibility. Within the 80% development set, we performed a non-nested 5-fold stratified group *K*-fold cross-validation, using the protein ID as the grouping variable to ensure that peptides from the same protein were always assigned to the same fold.

### Independent dataset

For the mouse RBP dataset, we sourced a total of 2,897 RBPs from the RBP2GO database [28]. To ensure independence, we excluded any RBPs already present in our training or testing datasets, resulting in a final independent dataset of 1,482 RBPs, which served as an unseen benchmark for real-world inference.

### External validation on the TEST474 dataset

To rigorously assess the specificity of caRBP-Pred, we performed an additional evaluation using the external dataset TEST474 [24]. The TEST474 dataset was categorized into 3 groups based on their verified binding affinities: proteins confirmed to bind neither DNA nor RNA (neither, *n* = 223), utilized as a rigorous negative control set to estimate the false positive rate; generic RBPs used to evaluate the model’s ability to distinguish caRBPs from general cytoplasmic or nucleoplasmic RBPs (RNA binding only, *n* = 68); and proteins experimentally verified to interact with both DNA and RNA (DNA and RNA binding, *n* = 8). The pLM–CNN–BiLSTM model was applied to this dataset without further fine-tuning.

### Prediction of protein and peptide SSs

To predict the SSs of proteins and peptides, we employed PS4 with default parameters [29]. This tool generated SS annotations corresponding to the primary amino acid sequences of each protein and peptide in our datasets.

### Feature representation and pLMs

We developed an end-to-end deep learning framework to predict protein types based on primary sequences and predicted SSs. Amino acids and SS states were transformed into numerical vectors using label encoding with the 20 standard amino acids mapped to integers [1, 20] and SS states to [1, 8] (0 reserved for padding masks).

To leverage advanced protein representations, we utilized the ProtT5-XL pre-trained transformer model [30]. High-dimensional embeddings (1,024-dimensional) were extracted for each residue and further processed via global average pooling to generate fixed-length vector representations for downstream classification.

### Model architecture and ablation study

To evaluate the predictive performance, we implemented and compared several state-of-the-art architectures (Table 1).

**Table 1.** Parameter configuration for model implementation

Models	Parameter settings
CNN–BiLSTM	embedding_dim = 64, LSTM_units = 64, CNN_filters = 64, kernel_size = 3, padding = “same”, activation = “relu”, AttentionLayer, GlobalAveragePooling
CNN	embedding_dim = 64, CNN_filters = 128, kernel_size = 3, activation = “relu”, GlobalAveragePooling
ResNet–CNN	embedding_dim = 64, initial_CNN_filters = 64, kernel_size = 7, residual_blocks = 3, GlobalAveragePooling
BiLSTM	embedding_dim = 64, LSTM_units = 64, return_sequences = True, AttentionLayer
RF	n_estimators = 100, random_state = 42
SVM	probability = True, random_state = 42
XGBoost	use_label_encoder = False, eval_metric = “logloss”, random_state = 42

CNN, convolutional neural network; BiLSTM, bidirectional long short-term memory; ResNet, residual network; RF, random forest; SVM, support vector machine

#### CNN–BiLSTM

**Fig. 1.**
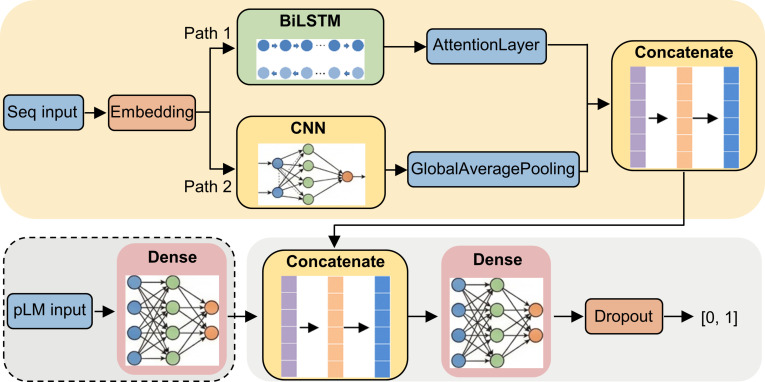
Architecture of the hybrid protein language model (pLM)–convolutional neural network (CNN)–bidirectional long short-term memory (BiLSTM) model for chromatin-associated RNA-binding protein (caRBP) prediction.

To capture both global contextual dependencies and local sequence motifs, we designed a hybrid CNN–BiLSTM architecture with an attention mechanism. The model processes the raw sequence input through an initial embedding layer, which is then fed into 2 parallel feature extraction branches. The first branch employs a BiLSTM network integrated with an attention layer to model long-range dependencies and highlight informative regions within the sequence. Simultaneously, the second branch utilizes a one-dimensional CNN followed by global average pooling to extract salient local patterns. For enhanced representation, features from a pLM can be optionally integrated via a dedicated dense layer. Finally, the fused feature vectors are passed through a fully connected multilayer perceptron head with dropout regularization for binary classification (Fig. 1).

#### ResNet–CNN

Residual network (ResNet)–CNN is a deep convolutional network employing residual blocks to mitigate the vanishing gradient problem and extract high-level hierarchical features.

#### Machine learning models

To establish a performance baseline for classical methods, we implemented random forest, SVMs, and XGBoost, using the encoded sequences as input features.

### Model training parameters

Models were implemented using TensorFlow/Keras and trained with the Adam optimizer. To prioritize the identification of the minority positive class (chromatin-contact peptides), we implemented a hybrid Dice–focal loss function. This function combined focal loss (with a focusing parameter *γ* = 2.0 and a weighting factor *α* = 0.9) with Dice loss to optimize the overlap between predicted and ground-truth distributions.

Training was conducted with the following specific configurations: A batch size of 32 was utilized for the sequence-based CNN–BiLSTM models, while a batch size of 512 was applied for models integrated with pLM features to optimize computational efficiency. Models were trained for a maximum of 30 epochs. An early stopping callback was implemented to monitor validation loss with a patience of 5 epochs, automatically restoring the best weights to prevent overfitting (Table 1).

### Motif enrichment analysis

To identify enriched motifs in the chromatin-contact peptide sequences, we utilized the XSTREME module from the MEME suite [31], with non-chromatin-contact peptide serving as the background. We specifically focused on motifs from the Eukaryotic Linear Motif database (version 2024) to provide biological context [32].

### Performance evaluation

We employed a 5-fold cross-validation procedure to rigorously evaluate our deep learning model. Performance metrics were calculated by averaging the model’s performance across the 5 validation sets. This approach ensured that our model’s predictive accuracy was robust and generalizable across different subsets of the data. The following metrics were used for quantitative assessment of the model’s performance:

$$\text{Accuracy}=\frac{\mathrm{TN}+\mathrm{TP}}{\mathrm{TN}+\mathrm{TP}+\mathrm{FP}+\mathrm{FN}}$$ (1)

$$\text{Precision}=\frac{\mathrm{TP}}{\mathrm{TP}+\mathrm{FP}}$$ (2)

$$\text{Recall}=\frac{\mathrm{TP}}{\mathrm{TP}+\mathrm{FN}}$$ (3)

$$F1=\frac{2*\text{precision}*\text{recall}}{\text{precision}+\text{recall}}$$ (4)

$$\mathrm{MCC}=\frac{\mathrm{TP}*\mathrm{TN}-\mathrm{FP}*\mathrm{FN}}{\sqrt{\left(\mathrm{TP}+\mathrm{FN}\right)\left(\mathrm{TP}+\mathrm{FP}\right)\left(\mathrm{TN}+\mathrm{FP}\right)\left(\mathrm{TN}+\mathrm{FN}\right)}}$$ (5)

The optimal model was selected through extensive architecture comparison, supported by statistical significance testing on metrics of average precision (AP), F1, and Matthews correlation coefficient (MCC).

### Evaluation of DRBP predictors

Since the source codes for previous DRBP predictors (iDRBP_MMC, DeepMC-iNABP, and DeepDRBP-2L) were not publicly available [24–26], we evaluated their accuracy on our training dataset using their provided web servers or executables.

### Model inference and prediction strategy

For proteome-wide identification of caRBPs, we developed a specialized inference pipeline. The prediction process follows a dual-input protocol to integrate primary sequence features with evolutionary information. To maintain consistency with the training phase, all protein sequences were standardized to a fixed length of *L* = 2,000 amino acids. Sequences exceeding this limit were truncated at the C-terminus, while shorter sequences were zero-padded to ensure a uniform input shape for the neural network. Residues were transformed into numerical indices, and residue-level pLM embeddings were loaded.

The inference was performed using the optimized pLM–CNN–BiLSTM hybrid model. To distinguish potential caRBPs from general RBPs with high confidence, we implemented a stringent classification threshold:

$$P\left(\text{caRBP}\right)>0.7$$ (6)

A protein was classified as positive only if its predicted probability exceeds 0.7; otherwise, it was designated as negative. This conservative threshold was chosen to prioritize specificity and minimize the false discovery rate during large-scale screening.

### Validation via the COMPARTMENTS and InterProScan databases

To verify the chromatin-binding potential of predicted caRBPs, we first utilized the COMPARTMENTS database (https://compartments.jensenlab.org/Search) to examine their subcellular localization. Candidates were screened using chromatin-related keywords, including *Chromatin*, *Nucleosome*, *Chromosome*, *Nucleus*, *Nucleoplasm*, *Nucleolus*, and *Nuclear matrix*. Furthermore, to identify functional DNA-binding domains (DBDs) within these potential caRBPs, we employed InterProScan (https://www.ebi.ac.uk/interpro/search/sequence/). Proteins were filtered for key regulatory domains and motifs, such as DBDs, zinc fingers, histone domains, chromatin domains, bromodomains, chromodomains, transcription factors, HMG, SKIP/SNW, homeoboxes, and PHD-fingers.

## Results

### Data composition and characteristics of sequences

**Fig. 2.**
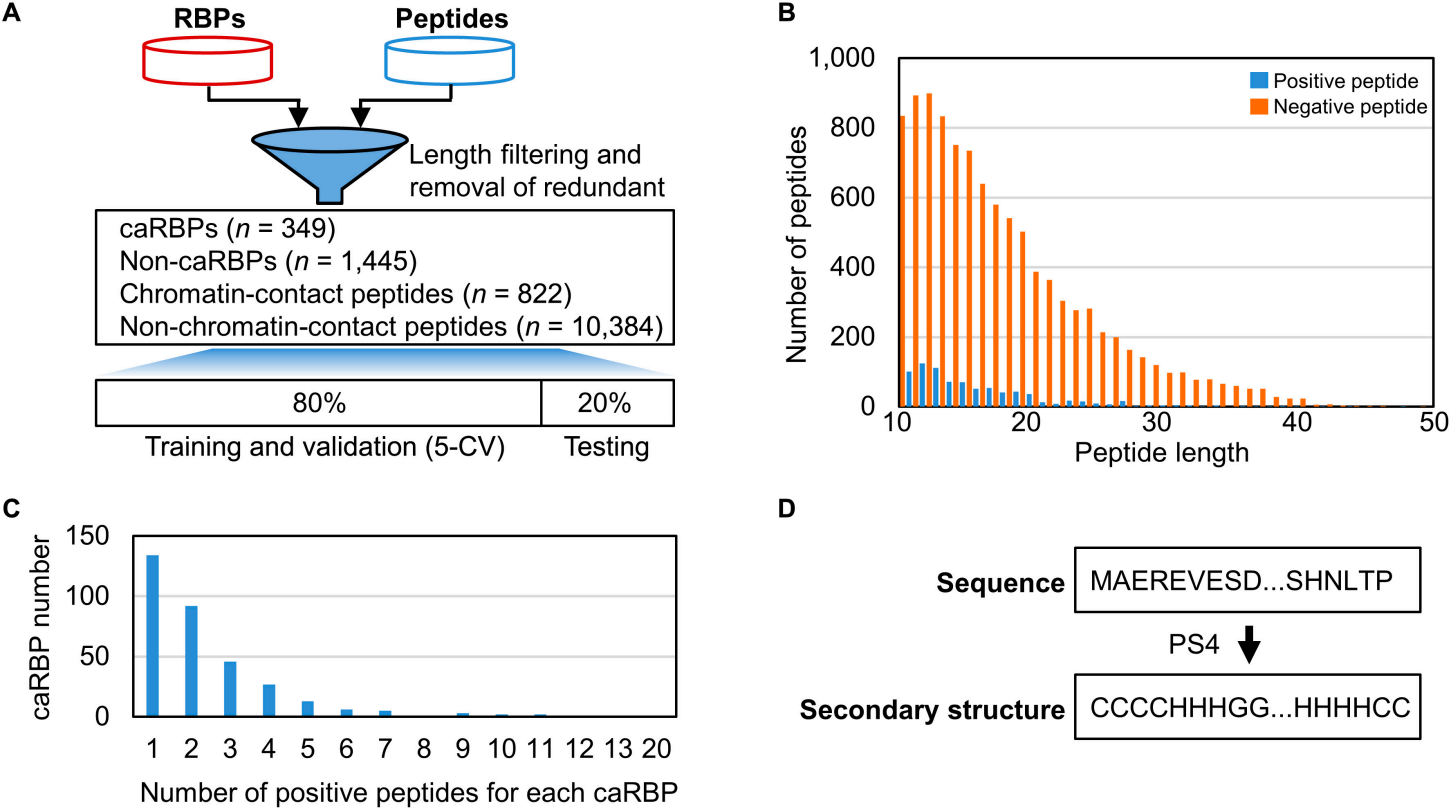
Data composition and processing. (A) Flowchart of the cleaning of raw data. (B) The distribution of peptide lengths was plotted. The distribution of protein lengths was not plotted because most protein lengths were unique (i.e., each length appears only once), resulting in a highly sparse frequency distribution. (C) Frequency of the number of chromatin peptides for each chromatin-associated RNA-binding protein (caRBP). (D) Flowchart of the prediction of secondary structure.

To predict caRBPs, we utilized a dataset of caRBPs and chromatin-contact peptides derived from mESCs [14]. After filtering and removing redundant sequences, we obtained a total of 349 caRBPs and 1,445 non-caRBPs for model development and evaluation (Fig. 2A). To characterize the differences between caRBPs and non-caRBPs, we employed cleverMachine to identify distinguishing physicochemical properties [33]. We found that nucleic-acid-binding ability effectively discriminated caRBPs from non-caRBPs, whereas other properties, including disorder propensity, aggregation, burial propensity, and hydrophobicity, did not show significant discriminative power (Fig. S1). These results suggest that nucleic-acid-binding ability reflects a core characteristic of caRBPs, supporting the feasibility of a deep-learning-based prediction approach.

Additionally, we compiled 822 chromatin-contact peptides (positive peptides) derived from caRBPs and 10,384 peptides (negative peptides) from RBPs lacking chromatin-binding ability (Fig. 2A). The peptide length distribution showed that the majority of positive peptides range from 10 to 20 amino acids, with a maximum length of 49 amino acids (Fig. 2B). Frequency analysis revealed that approximately 38% (134/349) of caRBPs contain only one positive peptide, suggesting that chromatin-binding ability can be determined by very short sequences (Fig. 2C). Therefore, we reasoned that positive peptide features could be effectively used for caRBP inference. To further investigate the intrinsic differences between positive and negative peptides, we performed motif enrichment analysis and identified several motifs enriched in positive peptides, including “.G[RK][RK]”, “.(S)..F.K”, and “...([ST])P[RK]” (Fig. S2). These motifs are associated with key chromatin-regulatory interfaces. Specifically, the .G[RK][RK] motif aligns with basic patches found in histone H3 tails that facilitate protein–protein interactions within the nucleosome. The .(S)..F.K motif is characteristic of ligands for BRCA1 C-terminus domains, which are essential for recruiting silencing complexes to damaged chromatin. In addition, the positive charges of R/K residues mediate electrostatic interactions with the DNA backbone [34], while glycine/arginine-rich motifs are known drivers of phase separation, facilitating the formation of protein–chromatin condensates [35,36]. These 2 mechanisms may explain the chromatin-binding ability of caRBPs.

Finally, we extracted primary amino acid sequences and predicted SS information as inputs for the model (Fig. 2D), as primary sequences alone may not capture spatial and conformational aspects critical for chromatin binding.

### CNN–BiLSTM outperforms other models for caRBP prediction

**Fig. 3.**
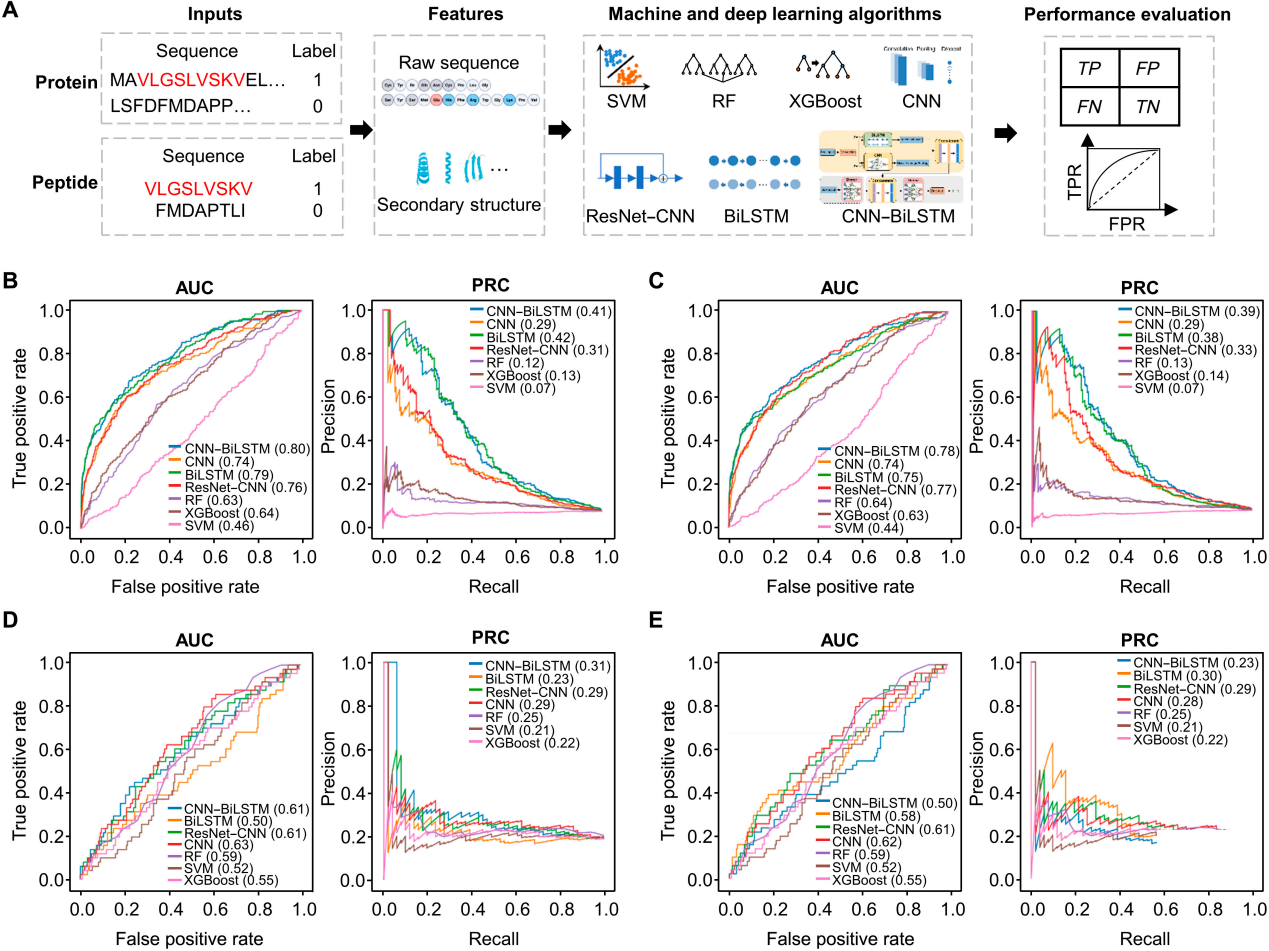
Convolutional neural network (CNN)–bidirectional long short-term memory (BiLSTM) outperforms other models for chromatin-associated RNA-binding protein (caRBP) prediction. (A) The flow diagram depicts the steps to develop the proposed caRBP prediction model. (B to E) Area under the curve (AUC; left panel) and precision–recall curve (PRC; right panel) plots of peptide sequences (B), combination of peptide sequences and secondary structure (C), protein sequences (D), and combination of protein sequences and secondary structure (E) on various models.

Given that CNN–BiLSTM architectures have been successfully implemented in various studies for predicting DNA-binding proteins, RBPs, and their binding sites [25,37–40], we developed a CNN–BiLSTM model alongside several baseline models, including CNN, ResNet–CNN, BiLSTM, SVM, random forest, and XGBoost, to identify the optimal approach for caRBP prediction (Fig. 3A).

For peptide-level inputs, the CNN–BiLSTM model consistently outperformed other models (Fig. 3B, Table 2, and Table S1). However, its performance on imbalanced datasets remained modest (AP = 0.41, F1 = 0.38, and MCC = 0.32) (Fig. 3B and Table 2), likely due to the limited number of positive peptide samples (*n* = 822) and high sequence similarity. Indeed, after removing redundant sequences at a 40% similarity cutoff, only 45 chromatin-contact peptides remained (data not shown). Adding SS information did not further improve performance and even led to a slight decline in key metrics (AP = 0.39, F1 = 0.35, and MCC = 0.30) (Fig. 3C, Table 2, and Table S1).

**Table 2.** Model performance for peptide classification on an independent testing dataset

Input type	Models	AUC	AP	F1	MCC	ACC	Prec	Recall
Peptide sequence only	CNN–BiLSTM	0.80	0.41	0.38	0.32	0.88	0.31	0.45
	CNN	0.74	0.29	0.32	0.26	0.83	0.22	0.52
	BiLSTM	0.79	0.42	0.38	0.31	0.87	0.32	0.50
	ResNet–CNN	0.76	0.31	0.33	0.26	0.87	0.27	0.40
	RF	0.63	0.12	0.00	0.00	0.92	0.00	0.00
	XGB	0.64	0.13	0.07	0.07	0.91	0.23	0.04
	SVM	0.46	0.07	0.00	0.00	0.92	0.00	0.00
Peptide sequence + secondary structure	CNN–BiLSTM	0.78	0.39	0.35	0.30	0.86	0.28	0.48
	CNN	0.74	0.29	0.30	0.25	0.79	0.20	0.55
	BiLSTM	0.75	0.38	0.33	0.28	0.84	0.24	0.51
	ResNet–CNN	0.77	0.33	0.32	0.26	0.84	0.23	0.48
	RF	0.64	0.13	0.00	0.00	0.92	0.00	0.00
	XGB	0.63	0.14	0.08	0.07	0.91	0.23	0.05
	SVM	0.44	0.07	0.00	0.00	0.92	0.00	0.00

AUC, area under the curve; AP, average precision; MCC, Matthews correlation coefficient; ACC, accuracy; Prec, precision; CNN, convolutional neural network; BiLSTM, bidirectional long short-term memory; ResNet, residual network; RF, random forest; XGB, XGBoost; SVM, support vector machine

When using full-length protein sequences, the CNN–BiLSTM model demonstrated competitive performance on the independent testing set, achieving an area under the curve (AUC) of 0.61 and an AP of 0.31 (Fig. 3D, Table 3, and Table S1). Interestingly, integrating SS information again provided no additional performance gain, with some metrics even exhibiting a marginal decline (AP = 0.23, F1 = 0.36, and MCC = 0.16) (Fig. 3E, Table 3, and Table S1). Considering all metrics, the sequence-only CNN–BiLSTM model using either peptides or full-length proteins was selected for further optimization.

**Table 3.** Model performance for protein classification on an independent testing dataset

Input type	Models	AUC	AP	F1	MCC	ACC	Prec	Recall
Protein sequence only	CNN–BiLSTM	0.61	0.31	0.37	0.18	0.43	0.24	0.88
	BiLSTM	0.50	0.23	0.31	0.00	0.18	0.18	0.98
	ResNet–CNN	0.61	0.29	0.33	0.09	0.37	0.21	0.84
	CNN	0.63	0.29	0.33	0.10	0.47	0.22	0.84
	RF	0.59	0.25	0.00	0.00	0.81	0.00	0.00
	SVM	0.52	0.21	0.00	0.00	0.81	0.00	0.00
	XGBoost	0.55	0.22	0.00	0.00	0.80	0.00	0.00
Protein sequence + secondary structure	CNN–BiLSTM	0.50	0.23	0.36	0.16	0.31	0.19	0.98
	BiLSTM	0.58	0.30	0.32	0.04	0.24	0.19	0.94
	ResNet–CNN	0.61	0.29	0.34	0.13	0.36	0.21	0.90
	CNN	0.62	0.28	0.34	0.14	0.47	0.24	0.84
	RF	0.59	0.25	0.00	0.00	0.81	0.00	0.00
	SVM	0.52	0.21	0.00	0.00	0.81	0.00	0.00
	XGBoost	0.55	0.22	0.00	0.00	0.80	0.00	0.00

AUC, area under the curve; AP, average precision; MCC, Matthews correlation coefficient; ACC, accuracy; Prec, precision; CNN, convolutional neural network; BiLSTM, bidirectional long short-term memory; ResNet, residual network; RF, random forest; SVM, support vector machine

### The pre-trained pLM substantially enhances caRBP prediction for full-length sequences

**Fig. 4.**
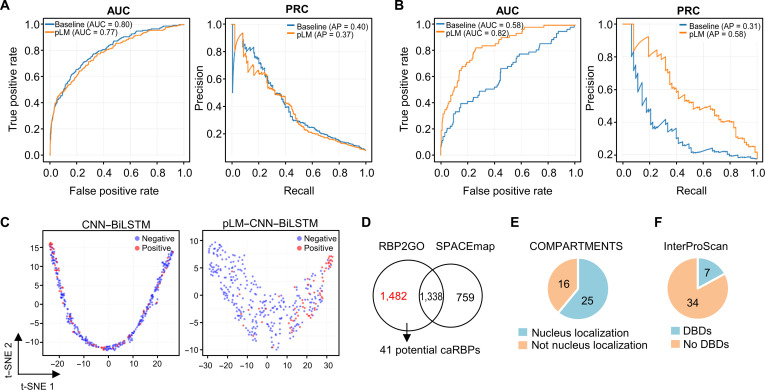
caRBP-Pred identifies potential chromatin-associated RNA-binding proteins (caRBPs) in the mouse. (A and B) Area under the curve (AUC) and precision–recall curve (PRC) plots of peptide sequences (A) and protein sequences (B) on the convolutional neural network (CNN)–bidirectional long short-term memory (BiLSTM) (baseline) or protein language model (pLM)–CNN–BiLSTM (pLM) model. (C) Feature visualization of the CNN–BiLSTM (left panel) and pLM–CNN–BiLSTM (right panel) models on proteins by t-distributed stochastic neighbor embedding (t-SNE) for dimension reduction. (D) The Venn diagram shows the overlap between mouse RNA-binding proteins (RBPs) from the RBP2GO database and training and testing datasets from SPACEmap. A total of 1,482 mouse RBPs were used for caRBP prediction, and 41 out of them were predicted as potential caRBPs. (E) Pie chart showing the number of nuclear-localized caRBP candidates. (F) Pie chart showing the number of caRBP candidates with known DNA-binding domains (DBDs).

Recent advances in pLMs have significantly improved the prediction of protein functions and structures [20–22], which can effectively capture sequence context, evolutionary information, and other critical features through training on millions of protein sequences. To enhance our model, we integrated ProtT5-XL, which was pre-trained on UniRef50 [30], with the CNN–BiLSTM framework, generating the pLM–CNN–BiLSTM framework. For peptide-level inputs, the pLM–CNN–BiLSTM model showed no significant improvement (AUC = 0.77, AP = 0.37, F1 = 0.32, and MCC = 0.34) over the baseline CNN–BiLSTM (AUC = 0.80, AP = 0.40, F1 = 0.28, and MCC = 0.35) (Fig. 4A, Fig. S3A and B, Table 4, and Table S2), possibly due to reduced sequence diversity, as the use of CD-HIT with an 80% similarity threshold may have resulted in highly similar sequences remaining in the dataset. In contrast, pLM–CNN–BiLSTM significantly improved performance on full-length protein sequences (AUC = 0.83, AP = 0.58, F1 = 0.52, and MCC = 0.41) compared to the baseline CNN–BiLSTM (AUC = 0.58, AP = 0.31, F1 = 0.32, and MCC = 0.19) (Fig. 4B, Fig. S3C and D, Table 5, and Table S2). Further feature visualization via t-distributed stochastic neighbor embedding showed that while the baseline model struggled to distinguish caRBPs and non-caRBPs, pLM–CNN–BiLSTM produced clear discrimination between the 2 classes (Fig. 4C). These results demonstrate that incorporating a pre-trained pLM significantly enhances the representational power of the model, particularly for full-length protein sequences.

**Table 4.** Average performance metrics of CNN–BiLSTM and pLM–CNN–BiLSTM for peptide classification on an independent testing dataset. Boldface is used to highlight the best-performing model and its corresponding metrics in each comparison group for easy identification by readers.

Models	AUC	AP	F1	MCC	ACC	Prec	Recall
CNN–BiLSTM (peptide)	**0.80**	**0.40**	0.28	**0.35**	**0.93**	**0.82**	0.17
pLM–CNN–BiLSTM (peptide)	0.77	0.37	**0.32**	0.34	0.93	0.64	**0.21**

CNN, convolutional neural network; BiLSTM, bidirectional long short-term memory; pLM, protein language model; AUC, area under the curve; AP, average precision; MCC, Matthews correlation coefficient; ACC, accuracy; Prec, precision

**Table 5.** Average performance metrics of CNN–BiLSTM and pLM–CNN–BiLSTM for protein classification on an independent testing dataset

Models	AUC	AP	F1	ACC	MCC	Prec	Recall
CNN–BiLSTM (protein)	0.58	0.31	0.32	**0.77**	0.19	0.34	0.30
pLM–CNN–BiLSTM (protein)	0.83	0.58	0.52	0.76	0.41	0.40	0.73

CNN, convolutional neural network; BiLSTM, bidirectional long short-term memory; pLM, protein language model; AUC, area under the curve; AP, average precision; MCC, Matthews correlation coefficient; ACC, accuracy; Prec, precision

### Specificity evaluation and external validation of the pLM–CNN–BiLSTM model

To further assess the specificity of pLM–CNN–BiLSTM, we evaluated its performance on an independent external validation dataset, TEST474 (Table 6). Our model achieved perfect specificity in the nonbinding group, yielding zero false positives (0/223). Among 68 proteins annotated as “RNA-binding only”, only 5 candidates (F1Q8J0, A2XKG2, A0A0A7HF73, Q91VU7, and Q9W0S7) were predicted as potential caRBPs, corresponding to a false positive rate of 7%. This low percentage is biologically consistent with the fact that caRBPs represent a highly specialized functional subset of the total RBP pool. Most canonical RBPs function primarily in the cytoplasm or nucleoplasm without direct chromatin association. The 5 potential caRBP candidates may represent previously unrecognized caRBPs rather than true false positives, given the frequent incompleteness of current functional annotations, which often overlook secondary chromatin-binding activities in generic RBP databases. A detailed domain analysis provides structural support for these predictions. For example, Q9W0S7 contains a staphylococcal nuclease-like (SNase) domain and an OB-fold, both known for nucleic acid interactions and nuclease activity [41,42]. Similarly, F1Q8J0 harbors an RRM motif, which is primarily known for RNA binding, has been shown to mediate interactions with single-stranded DNA in various chromatin-associated biological processes [43–45]. These findings suggest that the pLM–CNN–BiLSTM model, by leveraging pLMs, may have captured the subtle structural features of these dual-affinity proteins that are frequently missed by conventional sequence-alignment-based annotation methods.

**Table 6.** Performance of pLM–CNN–BiLSTM on an external protein dataset (TEST474)

Protein category	Sample size	Predicted caRBP	Predicted non-caRBP	False positive rate
Neither	223	0	223	0%
RNA binding only	68	5	64	7.3%
DNA and RNA binding	8	0	8	0%

pLM, protein language model; CNN, convolutional neural network; BiLSTM, bidirectional long short-term memory; caRBP, chromatin-associated RNA-binding protein

### Evaluation against existing DRBP predictors

We next examined whether existing DRBP predictors (iDRBP_MMC, DeepMC-iNABP, and DeepDRBP-2L) could accurately identify the caRBPs used in our training set. Since the source code for these tools is not publicly available, a direct head-to-head comparison under identical conditions was not possible. Instead, we evaluated their generalizability on our dataset. Despite performing well on their original datasets, all 3 tools showed limited recall on our caRBP training dataset (Table S3), as iDRBP_MMC recovered only 9 proteins, DeepMC-iNABP recovered 34, and DeepDRBP-2L recovered 75. These results highlight the limitations of current DRBP predictors for caRBPs and underscore the need for a specialized caRBP prediction tool.

### caRBP-Pred identifies potential caRBPs in the mouse

Using the optimized pLM–CNN–BiLSTM model, we developed caRBP-Pred and applied it to a curated set of 1,482 mouse RBPs. This analysis identified 41 high-confidence candidates as potential caRBPs (Fig. 4D and Table S4). Notably, core histone variants (e.g., H2ax and H3f3a) and transcription-related factors (e.g., Polr2e and Alyref2) yielded high confidence scores, consistent with their dual affinity for both RNA and the chromatin template. Additionally, several helicases such as Ddx55 were predicted as caRBPs, suggesting that they may act as molecular bridges that stabilize RNA–DNA–protein complexes. These results indicate that a subset of our predicted caRBPs may regulate gene expression both post-transcriptionally and through direct chromatin interaction.

To further characterize those potential caRBPs, we sought evidence from the COMPARTMENTS web server [46], which integrates the literature, high-throughput screens, and text mining. We found that 61% (25/41) of the predicted caRBPs showed strong chromatin-related localization annotations (e.g., Chromatin, Nucleosome, and Chromosome) with high confidence scores (Fig. 4E and Table S5). Furthermore, InterProScan [47] analysis revealed that approximately 17% (7/41) harbor well-characterized DNA-binding or histone-related domains, such as DBDs, zinc-finger domains, bromodomains, and HMG-box (Fig. 4F and Table S5). Altogether, these results demonstrate that caRBP-Pred, a high-performance framework for caRBP prediction, can reliably identify biologically plausible caRBPs, providing a valuable, cost-effective resource for exploring the chromatin-related functions of caRBPs.

## Discussion

Despite the widespread adoption of numerous computational methods for predicting general RBPs, a specialized framework for caRBPs has been lacking. Our study introduces caRBP-Pred, the first computational predictor specifically optimized to identify mouse caRBPs from full-length protein sequences. The competitive performance of caRBP-Pred compared to those of generic DRBP predictors underscores the unique sequence signatures of caRBPs, which often associate with chromatin through mechanisms beyond simple nucleic acid sequence recognition, such as protein–protein interactions and phase separation.

A key finding of this research is the transformative impact of integrating the pre-trained pLM ProtT5-XL into the CNN–BiLSTM framework. The pLM-augmented model demonstrated a substantial performance leap (AP = 0.58) over the baseline sequence-only model (AP = 0.31). This improvement is particularly critical for tasks with limited experimental datasets, as pLMs leverage evolutionary information and biophysical contexts learned from millions of sequences during pre-training. Our results suggest that while local motifs are important, the global linguistic context provided by pLMs is essential for capturing the multifaceted signatures required for chromatin association.

Although peptide-level models showed limited predictive power due to sample sparsity and high sequence similarity, motif enrichment analysis revealed biologically meaningful patterns. Enriched motifs such as .G[RK][RK] and ...([ST])P[RK] highlight the importance of positively charged arginine (R) and lysine (K) residues, which likely facilitate electrostatic interactions with the negatively charged DNA phosphate backbone. Furthermore, these glycine/arginine-rich regions are hallmark drivers of liquid–liquid phase separation, suggesting that many caRBPs may function by forming protein–chromatin condensates to regulate transcription.

Our inference pipeline identified 41 high-confidence caRBP candidates in the mouse proteome. The high proportion of these candidates (61%) with established nuclear or chromatin localization in the COMPARTMENTS database reinforces the reliability of our model. The identification of classical DBDs and chromodomains in a subset of these proteins further validates their potential for direct chromatin interaction. Interestingly, several proteins flagged as potential false positives in the external TEST474 dataset may instead represent under-annotated proteins with chromatin-associated functions, such as in transcriptional regulation.

While caRBP-Pred offers a powerful screening tool, certain limitations persist. The current model is trained on a relatively small dataset of experimentally verified RNA-independent caRBPs, which may not fully represent the diversity of chromatin-binding mechanisms. Moreover, the lack of temporal-spatial and cell-type-specific information limits our ability to predict how these interactions change across different biological contexts. Future work may further integrate single-cell multi-omics data and experimental validation (e.g., through chromatin immunoprecipitation sequencing or imaging) to definitively confirm the regulatory roles of these newly predicted caRBPs.

## Data Availability

The datasets, source code, and user notes are available on GitHub (https://github.com/callmeracle/caRBP-Pred).
