# Supplementary material for: caRBP-Pred: Leveraging Protein Language Models for the Prediction of Chromatin-Associated RNA-Binding Proteins
Source: Comput Struct Biotechnol J. 2026 Jun 5;35(1):0060. doi: 10.34133/csbj.0060 (PMC13336236; doi:10.34133/csbj.0060)
Supplement: Supplementary 1 — Figs. S1 to S3 Tables S1 to S5 [file csbj.0060.f1.zip › Suppl_Info.docx]

**Inventory of Supplementary Information**

**1. Supplementary Figures**

Figure S1. Physico-chemical properties of caRBPs (positive) and non-caRBPs (negative) predicted by cleverMachine.

Figure S2. Motif enrichment for chromatin-contact peptides.

Figure S3. Training and validation curves of the CNN-BiLSTM and pLM-CNN-BiLSTM models.

**2. Supplementary Tables**

Supplementary Table S1. Performance metrice on 5-CV dataset without pLM.

Supplementary Table S2. Performance metrice on 5-CV dataset with pLM.

Supplementary Table S3. caRBP prediction from DRBP prediction tools.

Supplementary Table S4. Predicted potential mouse caRBPs.

Supplementary Table S5. Results from COMPARTMENTS and InterProScan.

**3. Supplementary Figures Legends**

**Supplementary Figure S1. Physico-chemical properties of caRBPs (positive) and non-caRBPs (negative) predicted by cleverMachine.** Bar plots were generated by the cleverMachine web server based on the input of caRBPs and non-caRBPs sequences.

**Supplementary Figure S2.** **Motif enrichment for chromatin-contact peptides.**

**Supplementary Figure S3. Training and validation curves of the CNN-BiLSTM and pLM-CNN-BiLSTM models.** Loss (left) and Accuracy (right) curves are shown for: **(A)** CNN-BiLSTM at the peptide level; **(B)** pLM-CNN-BiLSTM at the peptide level; **(C)** CNN-BiLSTM (baseline) at the protein level; **(D)** pLM-CNN-BiLSTM (final framework) at the protein level. The transition from (C) to (D) highlights that integrating pLM effectively stabilizes the training process and enhances feature extraction for full-length protein sequences, leading to superior generalization and reduced false-positive rates.
