# Supplementary figures and images for: caRBP-Pred: Leveraging Protein Language Models for the Prediction of Chromatin-Associated RNA-Binding Proteins
Source: Comput Struct Biotechnol J. 2026 Jun 5;35(1):0060. doi: 10.34133/csbj.0060 (PMC13336236; doi:10.34133/csbj.0060)

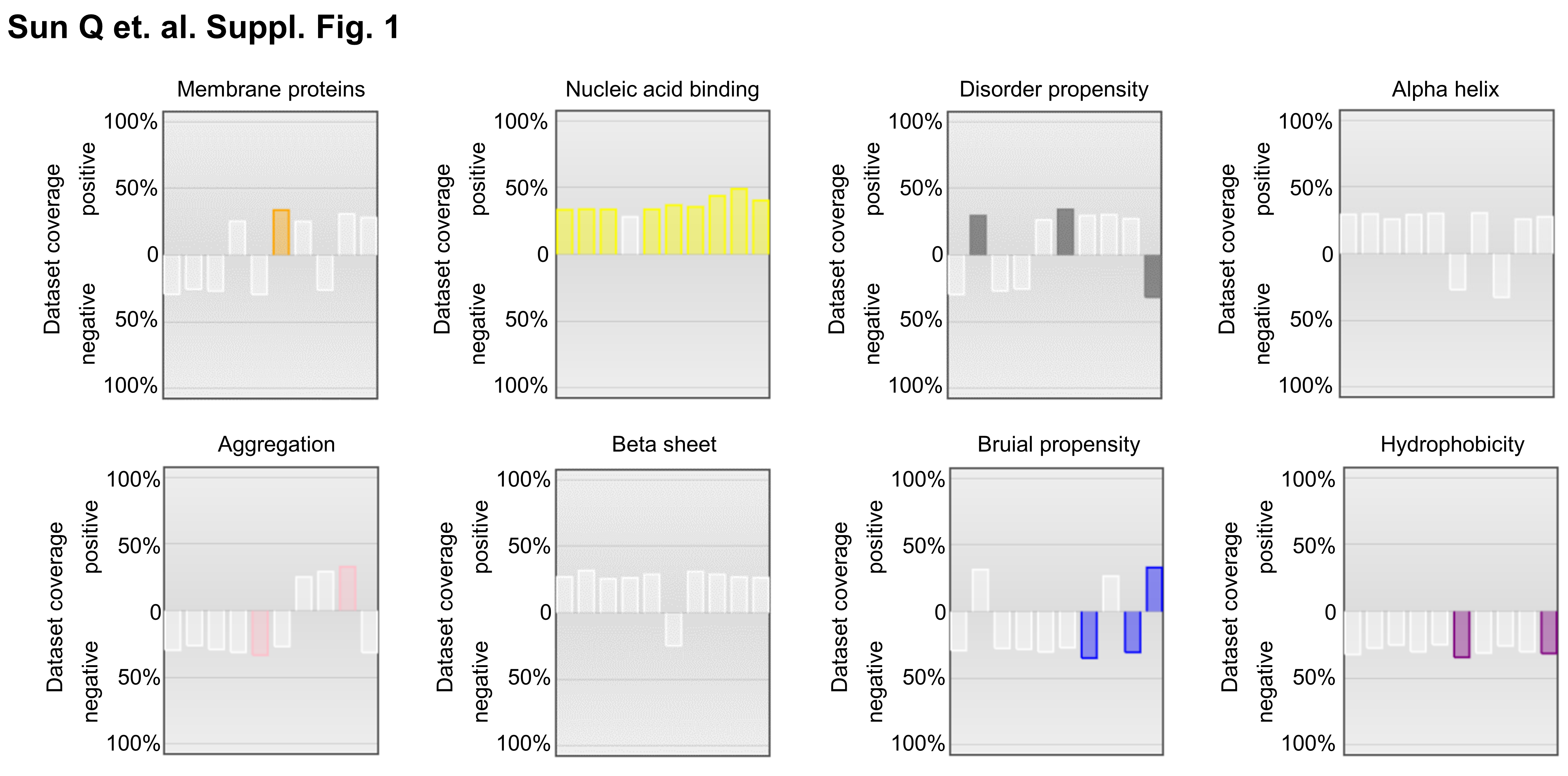

Supplement: Supplementary 1 — Figs. S1 to S3 Tables S1 to S5 [file csbj.0060.f1.zip › supp_figure1.tif]

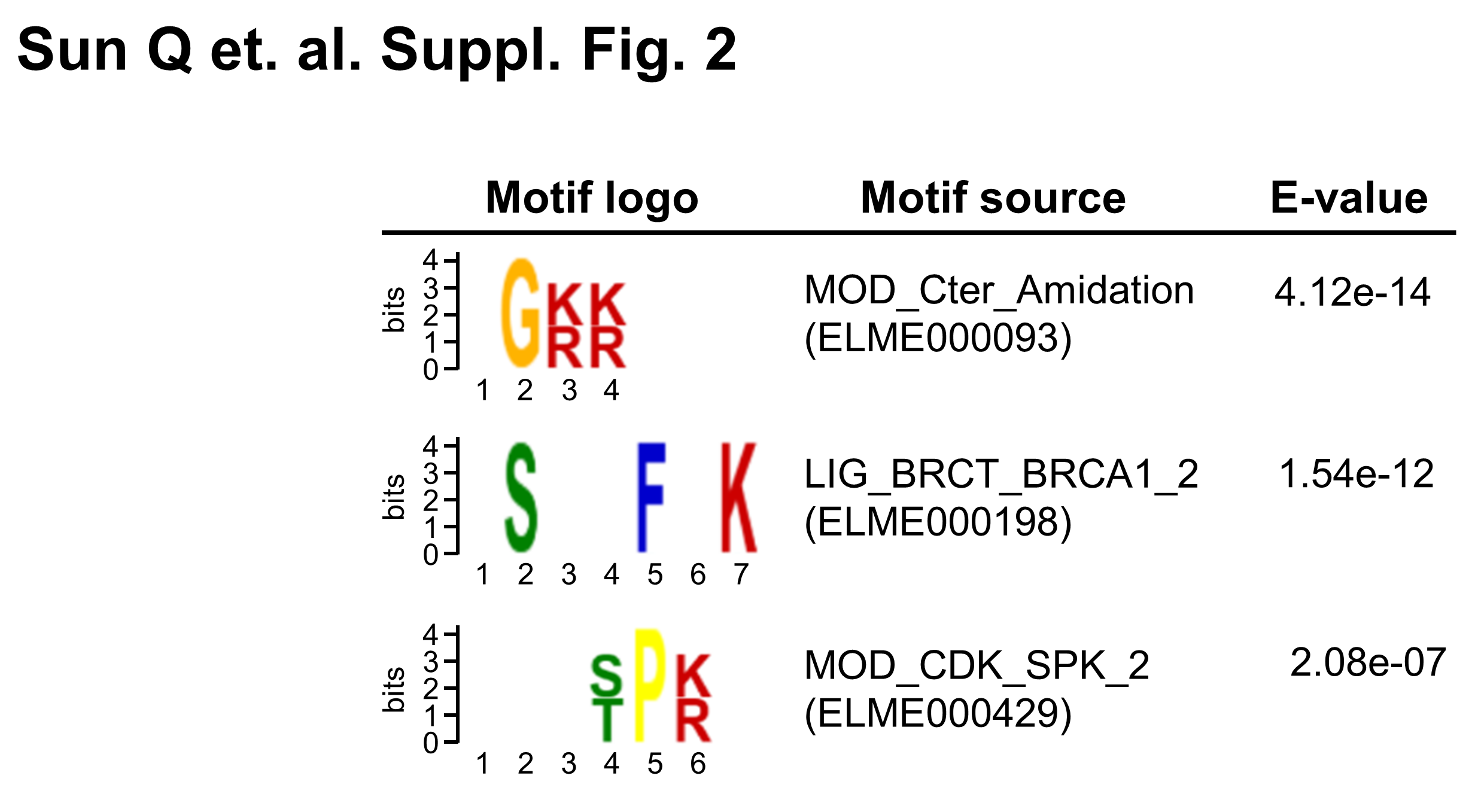

Supplement: Supplementary 1 — Figs. S1 to S3 Tables S1 to S5 [file csbj.0060.f1.zip › supp_figure2.tif]

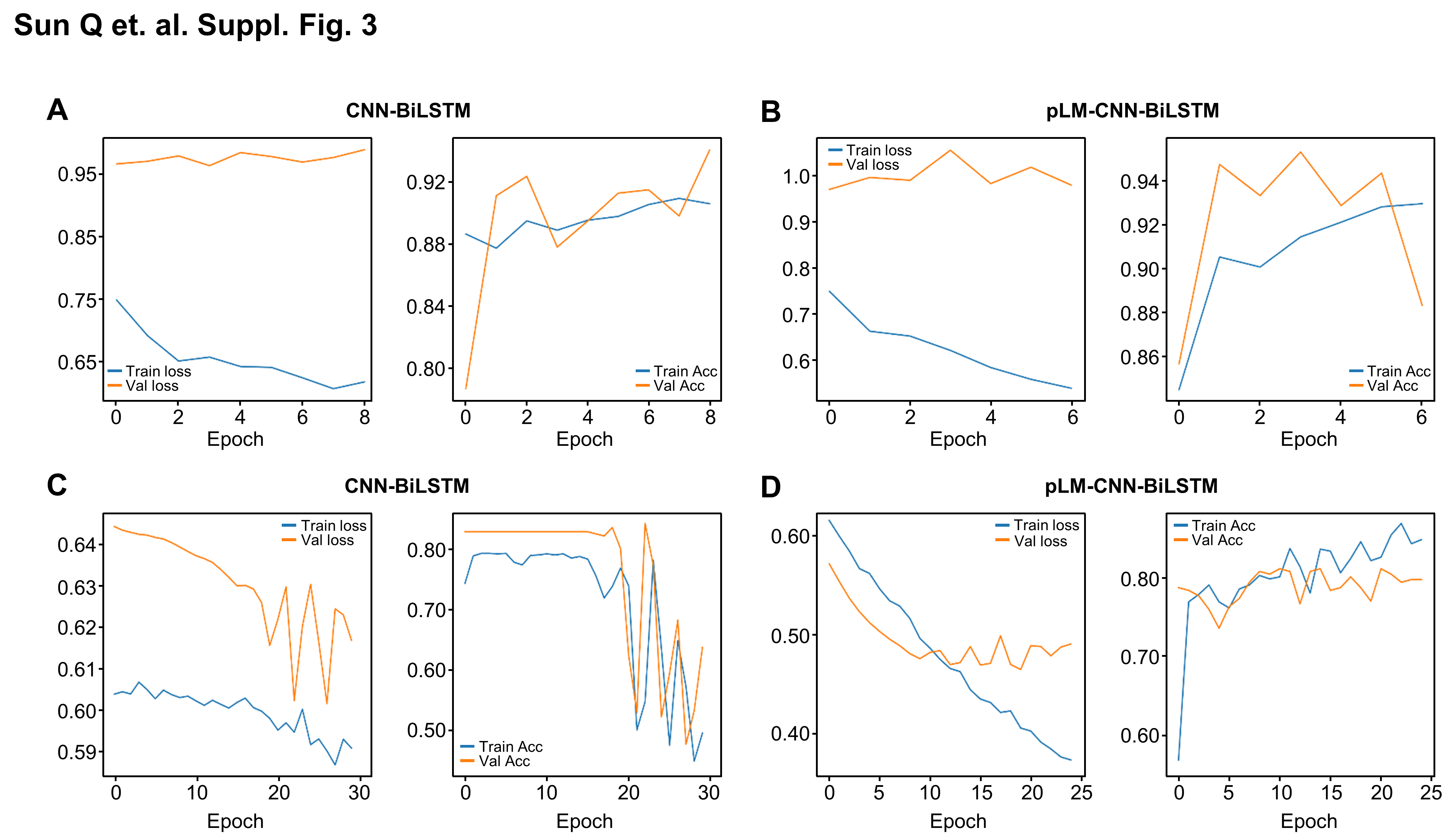

Supplement: Supplementary 1 — Figs. S1 to S3 Tables S1 to S5 [file csbj.0060.f1.zip › supp_figure3.tif]
